# Comparison of robotic and manual implantation of intracerebral electrodes: a single-centre, single-blinded, randomised controlled trial

**DOI:** 10.1038/s41598-021-96662-4

**Published:** 2021-08-24

**Authors:** Vejay N. Vakharia, Roman Rodionov, Anna Miserocchi, Andrew W. McEvoy, Aidan O’Keeffe, Alejandro Granados, Shahrzad Shapoori, Rachel Sparks, Sebastien Ourselin, John S. Duncan

**Affiliations:** 1 Department of Clinical and Experimental Epilepsy, Institute of Neurology, University College London, 33 Queen Square, London, WC1N 3BG UK; 2 National Hospital for Neurology and Neurosurgery, Queen Square, London, UK; 3 Chalfont Centre for Epilepsy, Gerrards Cross, UK; 4 Department of Statistical Science, University College London, London, UK; 5 School of Biomedical Engineering and Imaging Sciences, King’s College London, London, UK

**Keywords:** Epilepsy, Outcomes research, Biomedical engineering, Neurosurgery

## Abstract

There has been a significant rise in robotic trajectory guidance devices that have been utilised for stereotactic neurosurgical procedures. These devices have significant costs and associated learning curves. Previous studies reporting devices usage have not undertaken prospective parallel-group comparisons before their introduction, so the comparative differences are unknown. We study the difference in stereoelectroencephalography electrode implantation time between a robotic trajectory guidance device (iSYS1) and manual frameless implantation (PAD) in patients with drug-refractory focal epilepsy through a single-blinded randomised control parallel-group investigation of SEEG electrode implantation, concordant with CONSORT statement. Thirty-two patients (18 male) completed the trial. The iSYS1 returned significantly shorter median operative time for intracranial bolt insertion, 6.36 min (95% CI 5.72–7.07) versus 9.06 min (95% CI 8.16–10.06), *p* = 0.0001. The PAD group had a better median target point accuracy 1.58 mm (95% CI 1.38–1.82) versus 1.16 mm (95% CI 1.01–1.33), *p* = 0.004. The mean electrode implantation angle error was 2.13° for the iSYS1 group and 1.71° for the PAD groups (*p* = 0.023). There was no statistically significant difference for any other outcome. Health policy and hospital commissioners should consider these differences in the context of the opportunity cost of introducing robotic devices.

Trial registration: ISRCTN17209025 (https://doi.org/10.1186/ISRCTN17209025).

## Introduction

### Scientific background and rationale

Robotic surgical systems have seen exponential growth, both in the number of installations and breadth of procedures performed, over the last decade. The main applications have been in laparoscopic and thoracoscopic procedures^1^. Randomised control trials for some indications have not shown benefit for robotic-assistance over conventional laparoscopic surgery^2^. The economic cost of robotic surgery includes the purchase or lease of devices, associated instruments, consumables and service contracts, and increased utilisation of hospital resources including increased operating room time and staff training. Within the United Kingdom, the period 2011–2012 marked an inflexion point when the number of robotic prostatectomies exceeded both laparoscopic and open procedures for the first time^3^.

Comparatively, uptake of robotic systems for cranial neurosurgical procedures has been slow, despite early technological innovation and adoption^4^. The first robot-assisted stereotactic brain biopsy utilising a modified PUMA industrial robot was performed in 1985 with improved procedure time and intra-operative accuracy^5^. Global installations of stereotactic trajectory guidance systems increased in the last decade, with common cranial indications including brain biopsy^6^, deep brain stimulation^7^, stereoelectroencephalography (SEEG)^8,9^ and therapeutic drug delivery^10^.

SEEG is a procedure in which, usually, 7–16 electrodes are stereotactically implanted within predefined regions of the brain, to localize the source of drug-resistant focal epilepsy, in order to guide definitive resections to cure the epilepsy^9,11^. Recent years have seen a shift, particularly in North America, away from subdural grid placement towards SEEG due to the more favourable side effect profile of the latter^12^. Initially, SEEG electrodes were implanted using a stereotactic frame^13,14^. The restrictive, repetitive and time-consuming nature of frame-based approaches led to the development of frameless systems, which may give worse implantation accuracies^8^. Due to the number of electrodes implanted within individual patients and the requirement for precise implantation, SEEG has been suggested as being suitable for stereotactic robotic assistance. Despite the growth in the number and applications of robotic trajectory guidance systems, meta-analysis reveals that high-quality evidence supporting their use is limited^8^. To date, there are no reported prospective studies comparing robotic devices with manual frame-based or frameless systems.

This suggests novel robotic devices were introduced without parallel-group comparisons to the conventional methods. This may expose patients to increased harm until the associated learning curve is overcome and outcome accuracy related to the device can be compared.

We, therefore, undertook a randomised control trial of a novel robotic trajectory guidance device, the iSYS1 (Medizintechnik GmbH), to pragmatically assess the real-world consequences of implementing this technology in accordance with Stage 3 of the IDEAL framework for surgical innovation^15,16^.

### Specific objectives and hypotheses

The primary aim of this trial was to determine whether the iSYS1 robotic trajectory guidance system required less operative time for SEEG bolt insertion than the conventional frameless method utilising the precision-aiming device (PAD). Secondary aims were to identify effects on implantation accuracy, as well as gross differences in infection and intracranial haemorrhage rates.

## Methods

The study protocol was approved by the Health Research Authority on 20/02/2017, REC reference: 17/EE/0016 and the Medicines & Healthcare products Regulatory Agency, MHRA Reference: CI/2017/0026. The protocol was prospectively registered: ISRCTN17209025 on 14/11/2016, available online (https://doi.org/10.1186/ISRCTN17209025) and presented to key opinion leaders before recruitment^17,18^. No changes to the methods or design were made after the trial started.

### Trial design

A single centre, single-blinded randomised controlled trial of SEEG electrode implantation methods in patients with drug-refractory focal epilepsy reported in accordance with the CONSORT guidelines^19^.

### Participants

We included patients with drug-refractory focal epilepsy, due to undergo SEEG implantation as part of their pre-surgical evaluation, aged between 18–80 years and able to provide informed consent.

Exclusion criteria included pregnancy, uncorrectable coagulopathy, lack of capacity to consent and patients deemed unfit for general anaesthesia. Following a multi-disciplinary team discussion, all patients were given a patient information sheet before providing written informed consent. Written informed consent was taken by a delegated member of the research team and combined with a pre-operative hospital visit for digital subtraction angiography, 2–6 weeks before implantation. Patients were considered enrolled in the trial once randomised using Sealed Envelope.

### Interventions

Electrode trajectory planning was undertaken using the EpiNav™ platform^20,21^ before randomization to implantation method to prevent allocation bias, and the standard operating procedure was identical for each arm. In brief, EpiNav™ is a complex clinical decision support software for SEEG trajectory planning employing a semi-automated method based on user-defined parameters. Target and entry regions of interest are determined by a multi-disciplinary team of neurologists, neurophysiologists, neurosurgeons, neuropsychologists and neuropsychiatrists on the basis of non-invasive pre-surgical structural and functional MRI, PET, scalp video EEG, neuropsychological and psychiatric evaluations. EpiNav™ returns trajectories that minimise intracerebral length and drilling angle to the skull and maximises both absolute and cumulative distance from blood vessels (risk score) and grey-matter sampling. The algorithm ensures trajectories are > 10 mm from each other to prevent an intracranial collision. All plans were checked by a neurophysiologist and amended as appropriate by a neurosurgeon before implementation.

Ad-Tech (Oak Creek, WI) electrodes were used for SEEG and patients were randomised to insertion using either the PAD or the iSYS1 trajectory guidance system. All patients underwent insertion of bone fiducials under local anaesthesia for registration to the Medtronic S7 neuronavigation system. To minimise any confounding factors, the only difference in methodology between the two intervention arms was the device used for alignment of the drill guide to the pre-operatively planned trajectories.

**Figure 1:**
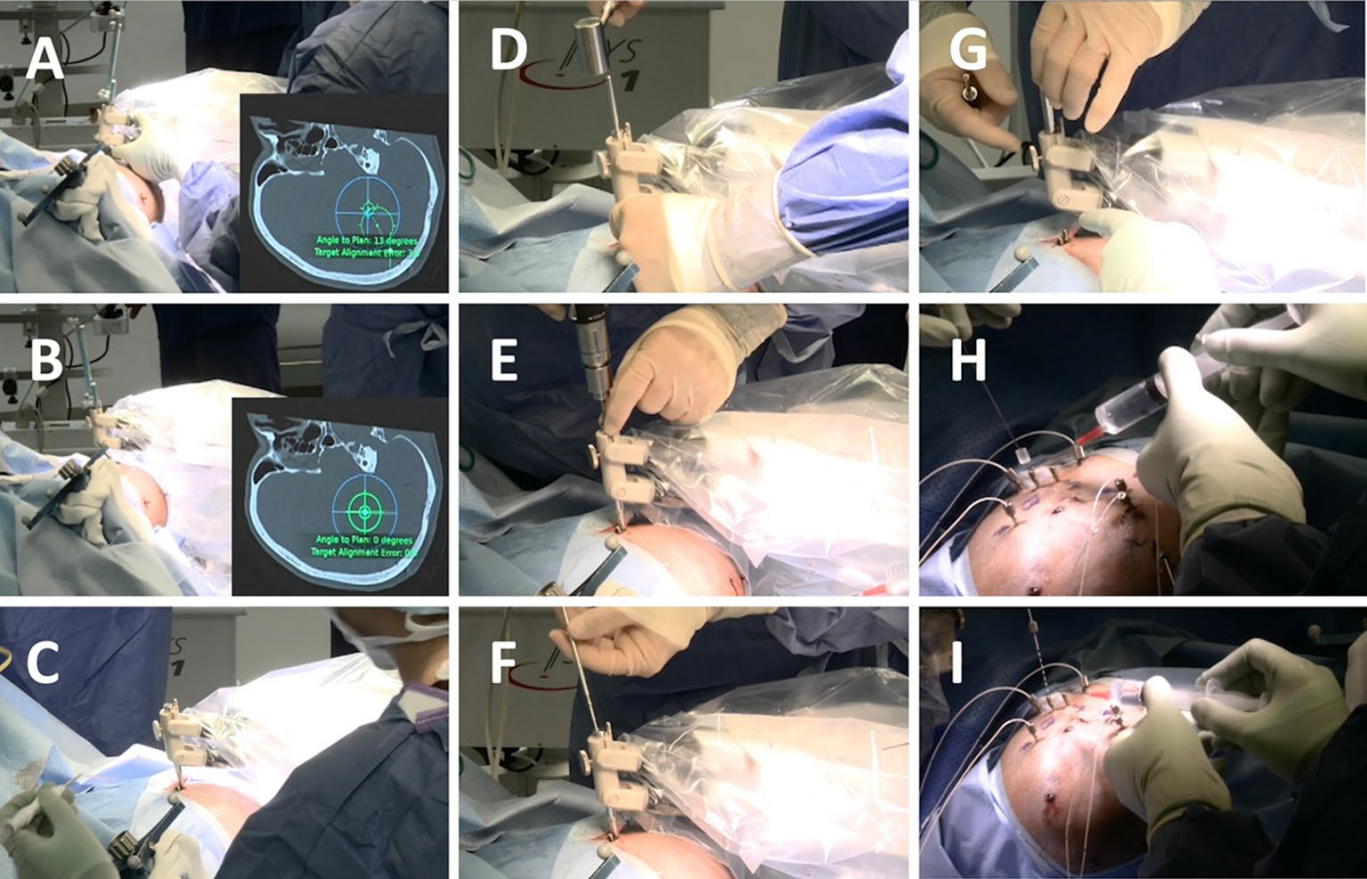
Steps for iSYS1 insertion of SEEG electrode. (**A**) The Vertek probe is inserted into the working channel of the iSYS1 device and the surgeon locks the device in rough alignment with the predefined trajectory. The Vertek probe is an optical navigation probe that once registered to the patient provides real world neuronavigation guidance. This is used to provide trajectory alignment accuracy information for both the iSYS1 and PAD implantation techniques. (**B**) The iSYS1 automatically aligns the working channel to the trajectory achieving an alignment accuracy of ≤ 0.1 mm to the target and 0 degrees to the plan. (**C**) The Vertek probe is removed from the working channel and replaced by a reduction tube. An incision is made in the skin to allow the reduction tube to contact the underlying skull. (**D**) A Steinmann pin is then inserted through the reduction tube and with gentle tapping a divot in the skull surface is made to define the entry point. (**E**) Drilling of the skull is performed through the reduction tube. (**F**) The skull anchor bolt is then fixed to the screwdriver and screwed into the skull through the reduction tube. (**G**) The reduction tube is removed from the working channel of the iSYS1 device to reveal the implanted bolt. (**H**) The iSYS1 device is then moved for insertion of the remainder of the bolts. After placement of all of the bolts, a stylet is placed through the bolt to the target point. (**I**) The electrodes are then placed through the bolts to the predefined depth.

The individual steps involved in each of these procedures is shown in Table 1 and Fig. 1. An image from a PAD implantation is shown in Supplementary Fig. 1.

**Table 1:** Operative steps associated with each implantation method.

Precision-aiming device	iSYS1 trajectory guidance system
1. Insertion of 6 bone fiducials under local anaesthesia	
2. CT scan	
3. General anaesthesia	
4. Placement of Mayfield Clamp	
5. Routine prep and drape	
6. Registration to S7 neuronavigation system with registration accuracy < 0.6 mm	
7. Freehand marking of entry points	
8. (A) Alignment of the precision-aiming device to first electrode trajectory	(A) Rough alignment of iSYS1 trajectory guidance system to a satisfactory position
(B) Achievement of trajectory with a target point accuracy of < 0.7 mm (current clinically accepted threshold)	(B) Precise alignment of iSYS1 trajectory guidance system to the final position with an accuracy < 0.1 mm (device threshold)
9. Skin incision at the defined entry point	
10. Steinmann pin used define entry point prior to drilling of trajectory	
11. Accuracy of trajectory checked with Vertek probe	
12. Insertion of the intracranial bolt	
13. Accuracy of trajectory checked with Vertek probe and new entry point set	
14. Removal of mechanical arm	
15. Measurement of electrode trajectory length (from top of the intracranial bolt to target point)	
16. Repeat steps 2–10 for each electrode to be inserted	
17. Insertion of the stylet to the predefined length derived from step 10	
18. Insertion of the electrode (Ad-Tech™) to the predefined length derived from step 10	
19. Repeat steps 12–13 for each electrode to be inserted	
20. Removal of bone fiducials	
21. Placement of sutures to close the incision

### Outcomes

The primary outcome was the operative time (minutes) for individual SEEG bolt insertion, defined as the time taken from the start of alignment to removal of the Vertek arm after bolt insertion. These steps were common to both techniques (see steps 2–9 of Table 1) providing systematic and objective time points.

Secondary outcomes included accuracy of SEEG electrode placement, the incidence of clinically and non-clinically significant haemorrhages, infection rate and new postoperative neurological deficit rate.

SEEG electrode placement accuracy measures were undertaken for the entry and target points using lateral deviation between the implemented and planned trajectory in an automated fashion. The algorithm segmented and reconstructed the individual electrodes based on the contacts identified from the post-operative CT as described in^22^ and returned all accuracy metrics in a blinded and automated fashion^23^. All segmentations were manually checked to ensure the correct contacts were assigned to the relevant electrodes. Entry point accuracies were back-projected and measured at the scalp surface. Due to the potential for the bolt to be displaced or bent following insertion, particularly in the temporal region, where the bone may be thin, or in patients with violent hypermotor seizures, we opted not to use the bolt axis to define the implemented trajectory. Instead, the most superficial contacts within the first 20 mm of the electrode were identified and a line of best fit was back-projected to the scalp surface to mark the implemented entry point of the electrode. The error in the angle of insertion was also determined based on the line of best fit.

Clinically and non-clinically significant haemorrhages were detected on postoperative neurological examination and post-operative imaging, respectively. All patients underwent a CT scan of the head immediately post-implantation and an MRI scan of the brain within 48 h. Radiological images were reported by a neuroradiologist blinded to the treatment arm allocation. Clinically significant haemorrhages were defined as those in which the patient had a postoperative complaint or neurological deficit and with a corresponding haemorrhage on the postoperative imaging. Non-clinically significant haemorrhages were defined as haemorrhages without any neurological consequence or clinical sequelae.

Clinical examination for neurological deficits was performed immediately postoperatively and at subsequent clinical interactions at 24 and 48 h. The electrode insertion sites were checked by the clinical teams, who were blinded to the implantation method, and any infection reported. All patients received prophylactic antibiotics for the duration of the SEEG implantation as part of institutional microbiology policy.

### Randomisation

We randomly assigned patients to the PAD or ISYS1 trajectory guidance implantation systems (using a 1:1 ratio), employing a computer-generated random sequence and random permuted blocks. An independent statistician created and tested the randomisation list which was then uploaded onto a computerised system provided by SealedEnvelope™. A designated member of the surgical team randomised patients by logging into the online system after a patient had given informed consent 2 to 7 days prior to the scheduled surgery date. No members of the trial team were aware of block sizes to ensure that allocation was concealed.

### Blinding

The patients, trial statistician and reporting radiologists were blinded to the intervention arm. For practical and logistical reasons it was not possible to blind the surgical and research team members.

### Sample size

The sample size was based on a difference of 20% in the median time for SEEG bolt insertion between the robotic and conventional frameless insertion groups, based on previously published data and our preclinical testing^24–26^. To detect this difference with a 5% significance level and power of 90% using a two-sample t-test on log-transformed insertion times would require 37 electrodes to be inserted in each group. This assumed that electrode insertion times have a log-normal distribution and that the median insertion time is approximately 20 min with a standard deviation of 5 min. In addition, we inflated the sample size to account for the clustering of electrodes within patients, assuming an estimated intraclass correlation coefficient of 0.2 and an average cluster size of 10 electrodes per patient. This would imply that 104 electrodes should be included in each group (approximately 11 patients per group assuming an average cluster size of 10 electrodes per patient). Finally, we increased this to a sample size of 16 patients per group to account for the possibility of patient drop-out and variable cluster size^27^.

### Statistical methods

All analyses used intention-to-treat principles (with patient data analysed by the group to which the patient was randomised). We analysed the electrode insertion time (minutes) using random-effects linear modelling to account for electrode clustering within patients with log-transformed times owing to right skewness in the insertion time distribution. We analysed electrode-level continuous secondary outcomes (skull entry point accuracy, target point accuracy and error of angle of implantation) using similar random effects linear models (with a log-transformation to account for skewness). Categorical secondary outcomes (numbers of haemorrhages, infections and neurological deficits) were summarised in tables by the randomised group. We performed all analyses using R (version 3.5.1).

### Role of the funding source

The funder of the study had no role in the study design, data collection or analysis and writing of the manuscript. External audit of trial data and procedures were performed at four stages during the trial including a closeout visit. In addition to the trial management group, trial steering and independent data monitoring committees were established for trial oversight. The corresponding author had full access to the data and has final responsibility for the decision to submit for publication.

## Results

### Recruitment

We recruited thirty-two patients from 21/10/2017 to 19/03/2019. All patients completed follow-up and were included in the quantitative analysis, with no switching between treatment groups. We randomly assigned 16 patients to the iSYS1 intervention (with 160 electrodes implanted) and 16 patients to the PAD group (with 168 electrodes implanted).

#### Baseline data

Baseline characteristics were evenly balanced between the two groups, although more males were assigned to the iSYS1 group (see Table 2).

**Table 2:** Demographic variables, stratified by randomised group.

Variable		Randomised group				
		iSYS1	PAD			
Sex	Male	12	6			
	Female	4	10			
Side of implantation	Left	8	8			
	Right	8	8			

Variable	Randomised group	No. of patients	Mean (SD)	Median	Minimum	Maximum
Age (years)	iSYS1	16	35.9 (8.2)	37.3	21.2	47.5
	PAD	16	32.5 (6.1)	33.4	22.0	42.0
Number of electrodes implanted	iSYS1	16	10.0 (1.6)	10.0	7	13
	PAD	16	10.5 (1.9)	10.5	7	14

#### Numbers analysed

See Supplementary Fig. 2.

### Outcomes and estimation

**Figure 2:**
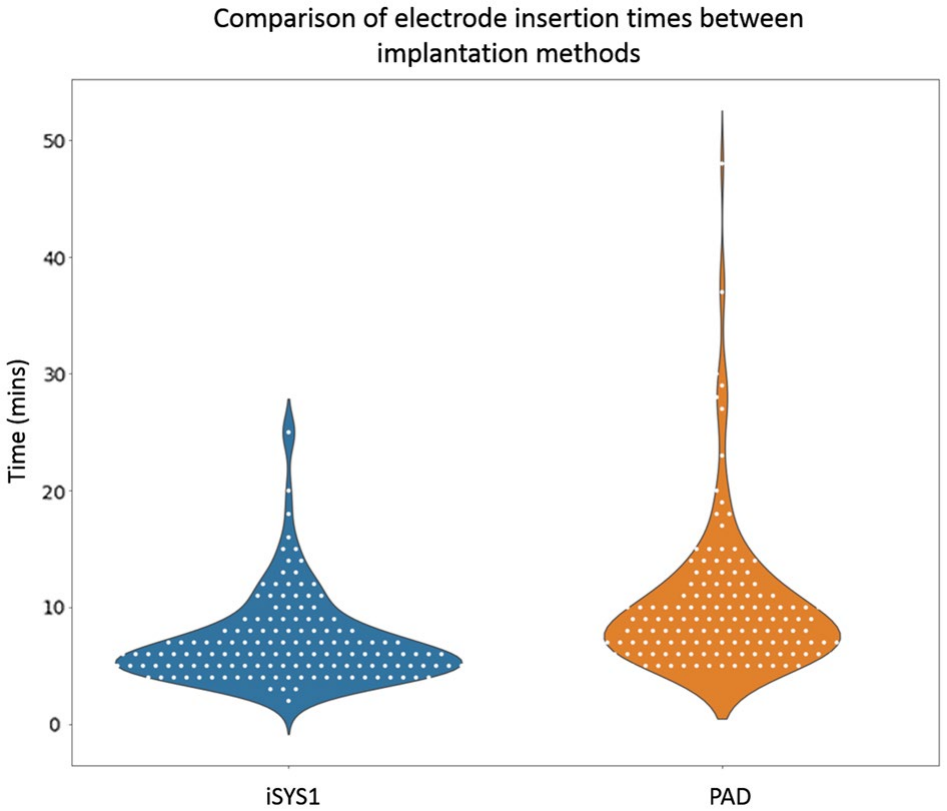
Combined violin and swarm plot demonstrating individual electrode insertion times (white dots) between the iSYS1 (blue) and PAD (orange) insertion techniques. The width of the violin plots correspond to the number of electrode insertions at each time point.

The primary outcome of individual bolt insertion implantation time for each electrode was significantly less for the iSYS group (median estimate of 6.36 min (95% CI 5.72 to 7.07) than for the PAD group (median estimate of 9.06 min (95% CI 8.16 to 10.06) *P* = 0.0001). We estimated the ratio of median insertion times (per electrode) for iSYS1 group/PAD group as 0.70 with 95% CI (0.61 to 0.81). This suggests an estimated reduction of insertion time per electrode of 30%, on average, for the iSYS1 group compared to the PAD group (see Table 3). The intraclass correlation (ICC) from the model for bolt insertion time was estimated as 0.15. See Fig. 2.

**Table 3:** Post-operative median estimates by group and between-group median ratio estimates (with 95% confidence intervals) from log-linear models fitted to the outcomes, assuming a log-normal distribution for outcome data.

	iSYS1 group	PAD group	Ratio of median estimate (iSYS1/PAD groups) (95% CI)	*P*-value**
Electrode insertion time (mins) Median (95% CI)	6.36 (5.72–7.07)	9.06 (8.16–10.06)	0.70 (0.61–0.81)	0.0001
Total operative time (mins)* Median (95% CI)	176.4 (153.7–202.6)	201.5 (175.5–231.3)	0.92 (0.78–1.08)	0.293
Entry point accuracy (mm) Median (95% CI)	1.09 (0.99–1.20)	1.17 (1.06–1.29)	0.93 (0.81–1.07)	0.334
Target point accuracy (mm) Median (95% CI)	1.58 (1.38–1.82)	1.16 (1.01–1.33)	1.37 (1.12–1.67)	0.004
Error of angle of implantation (degrees) Median (95% CI)	2.13 (1.87–2.41)	1.71 (1.51–1.94)	1.24 (1.04–1.48)	0.023
Post-operative haemorrhage	1/16 (6.25%)	2/16 (12.5%)		
Post-operative infection	0/16 (0%)	0/16 (0%)		
Post-operative neurological deficit	0/16 (0%)	0/16 (0%)		

Summary of post-operative outcomes by treatment group.

*Difference estimate adjusted by number of electrodes inserted. For electrode-level data, n = 160 for the iSYS1 group and n = 168 for the PAD group (with the exception of entry point accuracy, target point accuracy and angle error in the iSYS1 group where n = 159).

**P-values for a t-test of the null hypothesis of no difference in the corresponding outcome between groups, performed using log-transformed outcomes when fitting log-linear models.

Regarding secondary outcomes, electrode target point accuracy differed significantly between groups (median TPA estimates (95% CI) were 1.58 mm (1.38 to 1.82) and 1.16 mm (1.01 to 1.33) for the iSYS1 and PAD groups, respectively, P = 0.004). Electrode angle of implantation error differed significantly between groups (median angle error estimates (95% CI) were 2.13 degrees (1.87 to 2.41) and 1.71 degrees (1.51 to 1.94) for the iSYS1 and PAD groups, respectively, *P* = 0.023). Other secondary clinical outcomes, such as postoperative haemorrhage, infection and neurological deficit rate, did not differ significantly between groups (Table 3). Estimated median total operative time for the iSYS1 group was 176.4 min (95% CI 153.7 to 202.6) and that for the PAD group was 201.5 (95% CI 175.5 to 231.3). Noting that more electrodes were implanted in the PAD group (168 versus 160 in the iSYS1 group), an estimate of the ratio of median estimates (iSYS1 group/PAD group) with an adjustment for the number of electrodes inserted is 0.92 (95% CI 0.78 to 1.08, *P* = 0.29).

The entry point implantation accuracy of the iSYS1 was median 1.09 mm (95%CI 0.99 to 1.20) compared with 1.17 mm (95%CI 1.06 to 1.29) with the PAD and a ratio of median estimate (iSYS1/PAD groups) of 0.93 (95%CI 0.81 to 1.07), which did not reach statistical significance (*p* = 0.33, t-test on 30 D.O.F.).

As an early warning system to detect potential harm following the introduction of the novel medical device, we prospectively implemented a cumulative summation analysis. Subgroup analysis of the outcomes within chronological recruitment quartiles did not reveal any significant differences, suggesting that there was no detectable learning curve for the novel device^26^.

In 12 of 16 patients following robotic implantations, and 10 of 16 following manual PAD implantations a seizure onset zone was identified and resection recommended. In the remaining 4 of 16 patients following robotic implantations, 3 patients were ruled out of resective surgery following the SEEG and in one patient the seizure onset zone could not be adequately localised. From the 6 patients following manual PAD implantation that were not recommended for surgery, 4 patients were ruled out of resective surgery following the SEEG and in 2 patients the seizure onset could not be adequately localised.

### Harms

Adverse events related to SEEG insertion were noted in three patients, two of whom were in the PAD group. These were asymptomatic small volume haemorrhages detected on the immediate postoperative CT without clinical sequelae. The haemorrhages were located in subarachnoid space of the sylvian fissure following insular implantation, the subdural space and within the parenchyma at the cortical insertion site of an electrode. The haemorrhages were not related to electrode misplacement or inaccuracy and review of the pre-operative angiography did not reveal a vessel conflict when registered and fused to the post-implantation CT. As a result, these were not considered adverse device effects. There were no serious adverse events in either intervention arm of the trial.

## Discussion

The use of the iSYS1 robotic stereotactic trajectory alignment device significantly reduced SEEG bolt insertion time by 30%. Based on an average number of implanted electrodes of 10, this equated to a mean reduction in total operative time of around 30 min. The start of the total operative time was define as the time the patient arrived in the operating room from the adjacent anaesthetic room (following induction of the general anaesthesia) and finished at time the last suture was placed. As such, the total operative time included many other steps, including registration of the navigation system, positioning, draping and closure of the incisions (as shown in Table 1), and the overall difference in operative time was not statistically significant. This underlines the importance of reviewing the whole operative pathway to maximise efficiency, in order to realise the economic impacts of such a device. We highlight that total operative time is subtly different from the total anaesthesia time as we chose not to include the physician and patient specific factors that may affect the precise timing of induction of the general anaesthesia and removal of the endotracheal tube.

The target point and angle of insertion accuracy of the iSYS1, however, was significantly worse by an average 0.5 mm and 0.4 degrees, respectively. For cerebral SEEG implantations, these differences were not clinically relevant as this falls within the 3 mm safety margin applied during trajectory planning and all electrodes achieved their intended anatomical target structures based on the concept of ‘effective accuracy’^28^. There was no statistical difference in entry point accuracy between the two groups which is the most important safety metric, as the majority of blood vessels resulting in clinically significant haemorrhages are on the cortical surface^29^.

### Strengths and limitations

Strengths of this study include the comparison of a novel robotic device (iSYS1) that closely aligns with the current operative workflow and assigning the same surgeons to both intervention arms to mitigate bias. We employed a pragmatic study design in which we compared the conventional method of SEEG insertion which is established at the study institution with a novel robotic device that the surgeons had less experience of, reflecting the real-world adoption of intraoperative robotic technologies. We chose individual bolt insertion time as the primary outcome for the study due to the closely matched clinical workflow between the two implantation methods. As we show in Table 1, of the 21 steps within the study only the trajectory alignment step 8 differed between the two techniques. This provided the unique opportunity to focus the study on the disparity between the two techniques without the potential confounding nature of the common steps which may otherwise mask any significant differences and hence require a large sample size to reach significance. The main limitation of our study is that it is in a single centre. Due to the highly specialised nature of SEEG implantations, these procedures are concentrated at high volume epilepsy centres. We have previously ascertained that when a novel technique is introduced, this often supersedes the previous method without prospective parallel-group comparisons^8^. A multi-centre study would improve the sample size, generalisability and robustness of the results but would necessitate comparison to the method of current implantation used by the different surgeons and institutions.

Another potential limitation is the choice of robotic device. There are several different robotic devices available. We opted to compare the iSYS1 for a number of reasons. Firstly, the surgical workflow with the iSYS1 closely follows that of the frameless manual method that is currently used at our institution (see Table 1). This confers surgeon familiarity with the overall workflow associated with the novel device. This also allowed a more focused and direct comparison of the differences between the iSYS1 and the PAD arms, minimising confounding factors. Further, the iSYS1 system is compact and portable, with a small footprint, an established familiar interface through the Stealth station, relative cost and likely applicability of the findings to the stereotactic neurosurgical fraternity. Other robotic devices such as the NeuroMate and ROSA have not previously been compared to conventional stereotactic methods in a head to head prospective comparison. Typically, each surgical epilepsy centre generally has expertise with only a single robotic device, so we chose not to undertake a head-to-head comparison between robotic devices in the first instance. Finally, although we include postoperative haemorrhage, infection and neurological deficit rates within the secondary outcome for safety and surveillance considerations these should be considered exploratory in nature as the sample size was calculated based on the primary outcome and was, therefore, underpowered to detect such a difference. Based on an estimated symptomatic haemorrhage rate of 1 in 287 electrodes (1 in 29 patients)^30^, a prohibitively large sample size would be needed to show a comparative statistical difference. We report 1/16 (6.3%) asymptomatic (radiologically detected) haemorrhage in the PAD and 2/16 (12.5%) in the iSYS1 group. A recent report of a large case series from an established centre describing both frame-based and robotic methods revealed an asymptomatic haemorrhage incidence of 17%^31^.

### Generalisability

Robotic devices are becoming more established for stereotactic neurosurgical procedures with many high volume centres utilising them for SEEG, brain biopsy and deep brain stimulation procedures. Previous reports have prospectively confirmed the clinical workflow, utility and accuracy of the iSYS1 trajectory guidance system in accordance with Phase 2b of the IDEAL collaboration framework^24,25,32^. As wider acceptance of the devices occurs and centres consider their adoption, it is important that randomised control trials are undertaken to establish the comparative differences and whether these are sufficient to justify the cost. In accordance with Phase 3 of the Ideal collaboration, we compared the iSYS1 robotic device to the PAD frameless implantation system, but not to a frame-based system that would conventionally be considered the gold standard, as this reflects the standard of care at the study institution that has been performed since July 2012^33^. To optimize accurate trajectory alignment and safety of the PAD procedure, several iterative modifications and incremental improvements have been applied over a seven-year period, which has recently been reported^34^. These include the application of bone fiducials for registration to the neuronavigation system and use of ‘trajectory guidance’ during alignment (StealthStation S7 Cranial version 2.2.6 or later, Medtronic Inc.). To ensure implantation quality assurance, thresholds were set for image registration and electrode-trajectory alignment. Image registration utilising bone fiducials was applied to both arms of the study and only optical registration accuracies of < 0.6 mm were accepted. Image registration was repeated after draping of the patient and bone fiducials were left exposed during the procedure to allow registration to be checked and redone as necessary. ‘Trajectory guidance’ allows the alignment device, in this case, the Vertek probe, to align to both the entry and target points of the trajectory. This is in contrast to ‘target guidance’, which reports the alignment accuracy at the target point regardless of the cortical entry. The iSYS1 and the PAD were aligned according to the pre-operatively planned trajectories provided by the S7 neuronavigation system via the Vertek probe. Any systematic inaccuracy as a result of the optical registration or guidance would therefore equally affect both intervention arms. The accuracy of alignment to the pre-planned trajectory on the neuronavigation system for each electrode, regardless of the implantation method, had to achieve < 0.7 mm prior to commencement of drilling and bolt insertion. Due to the predefined accuracy threshold, implantation time was taken as the primary outcome measure between the two methods. An important distinction between the two intervention arms is platform stability. Both the PAD and the iSYS1 are fixed to the Vertek mechanical arm. During drilling any inadvertent lateral forces applied by the surgeon may result in slight displacement of the mechanical arm and Mayfield clamp, resulting in inaccurate angle of insertion and consequent target point error, but not the entry point. The PAD technique allows the trajectory alignment accuracy to be monitored continuously through the use of the SureTrak system. Deterioration in accuracy during the PAD technique would prompt the surgeon to realign before continuing with drilling. The iSYS1 did not have the same ability to detect this as continuous trajectory alignment accuracy measures were not possible during drilling and may be exacerbated by the moment introduced by weight of the device (1.2 kg). This may be one potential reason for the greater target point and insertion angle errors despite similar entry point accuracies.

### Interpretation

Before commencing the randomised control trial we undertook a PRISMA systematic review and mixed-effects meta-analysis of published SEEG methods and their corresponding accuracies^8^. The meta-analysis was registered on the PROSPERO database (registration number CRD42016047839), through which the review protocol can be accessed^35^.

Using the Preferred Reporting Items for Systematic Reviews and Meta-Analysis (PRISMA) guidelines^36^ we undertook a structured search of the PubMed, Embase and Cochrane databases. The last date of the search was undertaken on the 16/09/16. After applying eligibility criteria, 35 articles were subject to full manuscript review. A comparison of the articles for inclusion between the two independent researchers was undertaken and revealed high concordance between the identified studies. In total, 17 studies were included in the qualitative and 15 in the quantitative synthesis.

Following the completion of the trial, we have repeated the search criteria with the same systematic review methodology and updated the qualitative and quantitative analysis with subsequent manuscripts published after 16/09/16. The last date for the updated search was at the end of the trial (30/04/2019). A total of 524 publications were returned indicating an additional 196 additional publications during the trial period. After removal of non-English language and duplicate manuscripts, an additional 163 study title and abstracts were screened. Twenty-six manuscripts underwent full-text assessment and a further 3 studies were included in the quantitative synthesis. The remaining studies could not be included as they failed to reach the eligibility criteria or effects size and standard deviations were not provided. See Supplementary Fig. 3.

Supplementary Fig. 4: represents the PAD and iSYS1 entry (A) and target (B) errors as part of a random-effects meta-analysis of the published literature to date (last search 30/04/19). This suggests that both the PAD and iSYS1 arms returned accuracies similar to that of other robotic devices in the published literature. There was no overlap in the 95% confidence intervals of the PAD arm with any other studies in the frameless subgroup, suggesting that in the current study the PAD target point accuracy achieved was significantly greater than other studies in the reported literature. We hypothesise that this is due to the stringent accuracy criteria set during the alignment step and the continuous accuracy tracking during the drilling stage. Furthermore, the accuracy results achieved for the PAD arm are in keeping with our previously published metrics following years of incremental refinement^34^. This suggests that although the PAD results are more accurate than reports from other centres using frameless implantation methods, this is a consistent finding and not attributable to a ‘Hawthorn effect’ where the results may be artificially improved due to the awareness of being observed^37^. Based on the above results we intend to replace the use of the PAD with the iSYS1 trajectory guidance system at our institution for future SEEG implantations.

## Conclusion

This study shows that the iSYS1 robotic trajectory device reduces SEEG electrode implantation time compared to the frameless PAD device. The overall reduction in total operative time, however, that included positioning, draping, neuronavigation setup and closure was modest and not significantly different. For SEEG, the iSYS1 robotic guidance system offers a consistent and effective solution comparable to the PAD technique conventionally employed at our institution.

The entry and target point accuracies of both devices were similar to the reported results of other robotic devices such as the Neuromate (Renishaw, Gloucestershire, UK) and ROSA (Medtech, Montpellier, France) and were satisfactory for the placement of cerebral SEEG. They would also be suitable for placement of catheters and cerebral biopsies. Further research should focus on cost–benefit analyses of introducing robotic devices into clinical services and prospective head-to-head comparisons between different robotic devices in these and other indications such as DBS, where the accuracy differences may be more clinically relevant. 

## Supplementary Information

Supplementary Information 1.

Supplementary Information 2.

Supplementary Information 3.

Supplementary Information 4.

Supplementary Information 5.
