# Supplementary figures and images for: Comparison of robotic and manual implantation of intracerebral electrodes: a single-centre, single-blinded, randomised controlled trial
Source: Sci Rep. 2021 Aug 24;11:17127. doi: 10.1038/s41598-021-96662-4 (PMC8385074; doi:10.1038/s41598-021-96662-4)

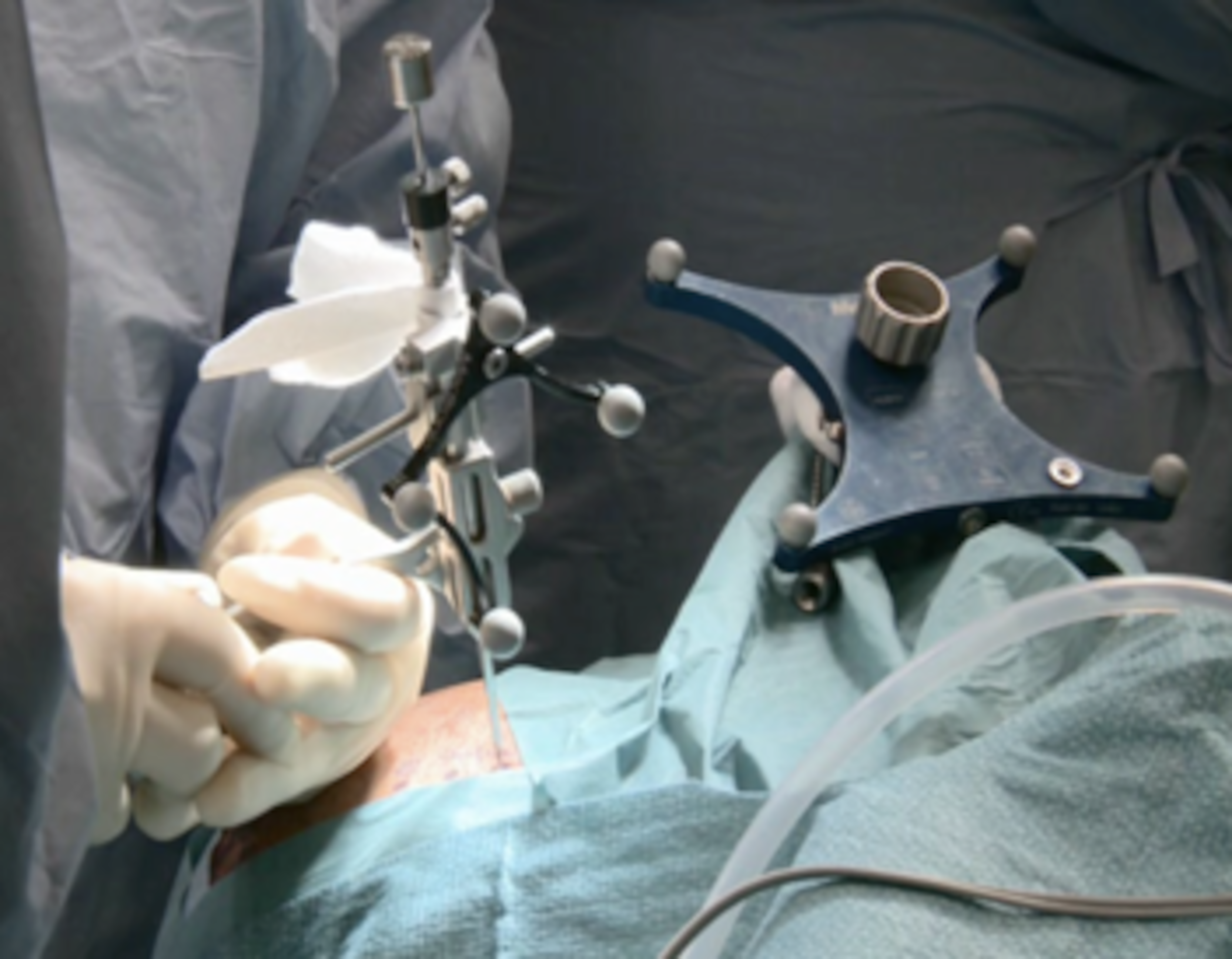

Supplement: Supplementary file 1 — Supplementary Information 1. [file 41598_2021_96662_MOESM1_ESM.tif]

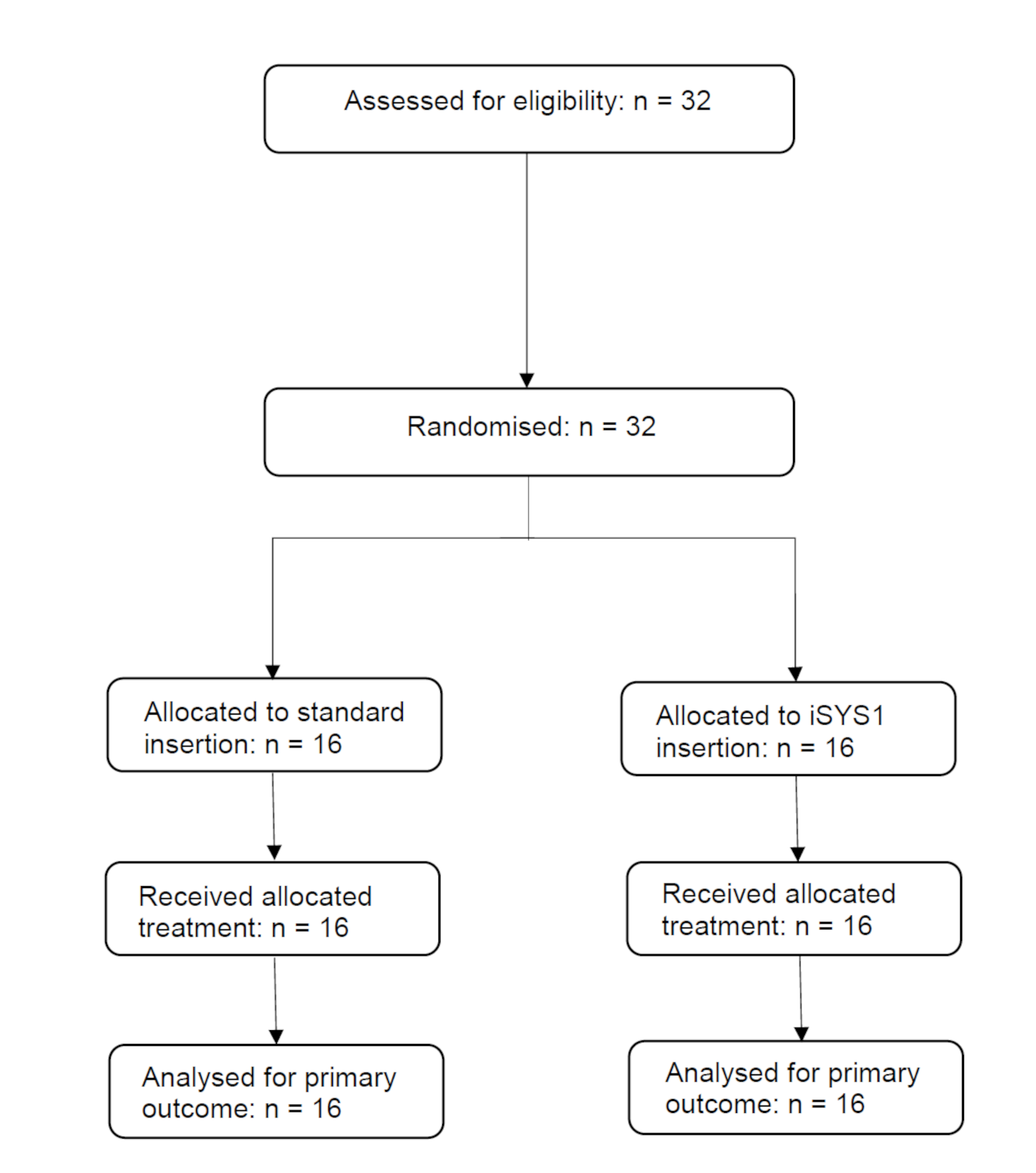

Supplement: Supplementary file 2 — Supplementary Information 2. [file 41598_2021_96662_MOESM2_ESM.tif]

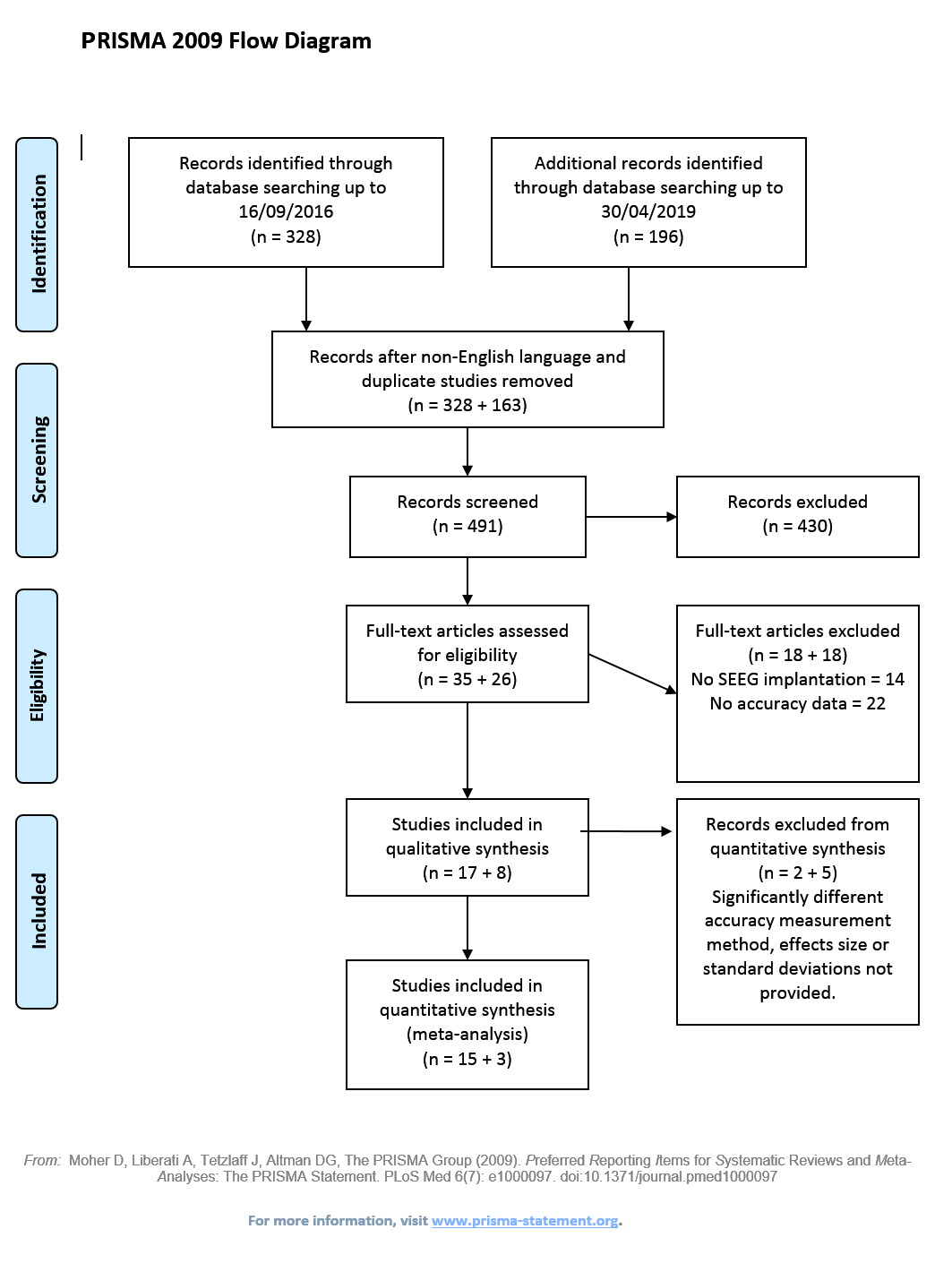

Supplement: Supplementary file 3 — Supplementary Information 3. [file 41598_2021_96662_MOESM3_ESM.tif]

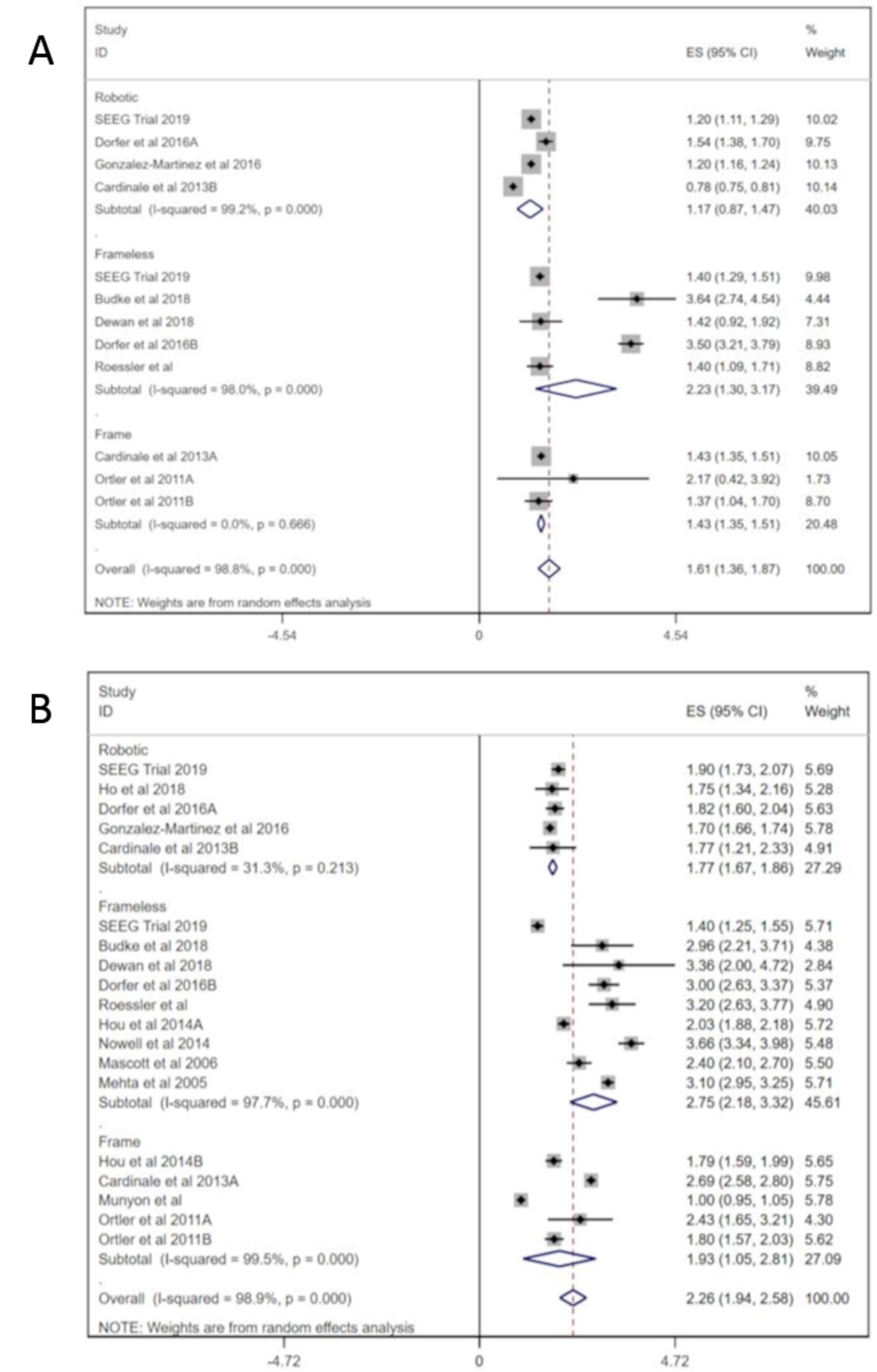

Supplement: Supplementary file 4 — Supplementary Information 4. [file 41598_2021_96662_MOESM4_ESM.tif]
