# Supplementary material for: Comparison of robotic and manual implantation of intracerebral electrodes: a single-centre, single-blinded, randomised controlled trial
Source: Sci Rep. 2021 Aug 24;11:17127. doi: 10.1038/s41598-021-96662-4 (PMC8385074; doi:10.1038/s41598-021-96662-4)
Supplement: Supplementary file 5 — Supplementary Information 5. [file 41598_2021_96662_MOESM5_ESM.docx]

Supplementary Figure 1 Legend: The PAD is held by the surgeon and the axis is aligned to the preplanned trajectory manually by the surgeon with the aid of the neuronavigation system. Once an acceptable trajectory alignment accuracy has been achieved, the mechanical arm is fixed in place and central working channel is then used for drilling and electrode insertion as outlined in the individual steps in Table 1.

Supplementary Figure 2 Legend: CONSORT diagram showing patient flow through each stage of the trial.

Supplementary Figure 3 Legend: PRISMA flow diagram showing search strategy and study inclusion in qualitative and quantitative synthesis from original (pre-trial) and updated (post-trial) meta-analysis.

Supplementary Figure 4 Legend: PAD and iSYS1 entry (A) and target (B) point errors as part of a random-effects meta-analysis. The vertical broken line represents an estimate of the overall median effect size when considering the robotic, frameless and frame-based techniques together. Note: SEEG Trial 2019 denotes the current study. (Study ID: Study Identifier).
